# Subcutaneous Basidiobolomycosis Mimicking Malignancy in an Immunocompetent Child: The Role of Multimodality Imaging and Targeted Biopsy

**DOI:** 10.7759/cureus.105762

**Published:** 2026-03-24

**Authors:** Deepshikha Arora, Avinash Dhok, Ashwini Umredkar, Chetana Ratnaparkhi, Shilpa Pande

**Affiliations:** 1 Department of Radiodiagnosis and Interventional Radiology, All India Institute of Medical Sciences, Nagpur, Nagpur, IND

**Keywords:** basidiobolomycosis, fungal infection, magnetic resonance imaging, subcutaneous mycosis, ultrasound-guided core biopsy, zygomycetes

## Abstract

Basidiobolomycosis is a rare subcutaneous fungal infection in immunocompetent children that frequently mimics inflammatory, vascular, and malignant soft-tissue pathologies on imaging. This is a diagnostically challenging case of a nine-year-old boy presenting with progressive, painless, diffuse subcutaneous swelling involving the right upper limb, chest wall, and neck. Ultrasound, contrast-enhanced magnetic resonance imaging (CE-MRI), and positron emission computed tomography (PET-CT) showed a diffuse, infiltrative soft-tissue lesion with increased metabolic activity, raising suspicion for a lymphoproliferative disorder or a slow-flow vascular malformation. Persistent eosinophilia and lack of response to antibiotics heightened diagnostic uncertainty. Definitive diagnosis was achieved only through ultrasound-guided targeted biopsy from the metabolically active component, which confirmed the diagnosis of subcutaneous basidiobolomycosis subsequently. The patient showed significant clinical improvement following itraconazole therapy. This case underscores basidiobolomycosis as an important radiological masquerader and highlights the pivotal role of multimodality imaging in directing precise biopsy and also in preventing misdiagnosis and unnecessary interventions.

## Introduction

Basidiobolomycosis is a rare, chronic subcutaneous fungal infection caused by *Basidiobolus ranarum*, a saprophytic fungus belonging to the order Entomophthorales of the class Zygomycetes [1]. The organism is widely distributed in tropical and subtropical regions and is found in soil, decaying vegetation, and the gastrointestinal tracts of amphibians, reptiles, and insects [1,2].

Unlike mucormycosis, basidiobolomycosis predominantly occurs in immunocompetent hosts, particularly children and young adults [3].

Clinically, subcutaneous basidiobolomycosis presents as a slowly progressive, firm, painless swelling involving the skin and subcutaneous tissues, most commonly affecting the extremities, trunk, buttocks, and chest wall [3,4]. Systemic symptoms are usually absent and non-specific, contributing to frequent diagnostic delay [2].

The indolent course and absence of constitutional symptoms often result in misdiagnosis as cellulitis, soft-tissue sarcoma, lymphoproliferative disease, and slow-flow vascular malformations. From a radiological perspective, basidiobolomycosis poses a significant diagnostic challenge due to its nonspecific imaging appearance and its ability to mimic malignant and vascular pathologies. Several reports describe cases initially interpreted as rhabdomyosarcoma, lymphoma, lymphangioma, and slow-flow vascular malformation on ultrasound, magnetic resonance imaging (MRI), and positron emission tomography (PET) scans [5,6].

Recognition of this entity and its characteristic imaging patterns is essential for radiologists, especially when evaluating immunocompetent patients in endemic regions, as it facilitates timely and appropriate tissue diagnosis while preventing misdiagnosis and unwarranted interventions.

## Case presentation

**Figure 1:**
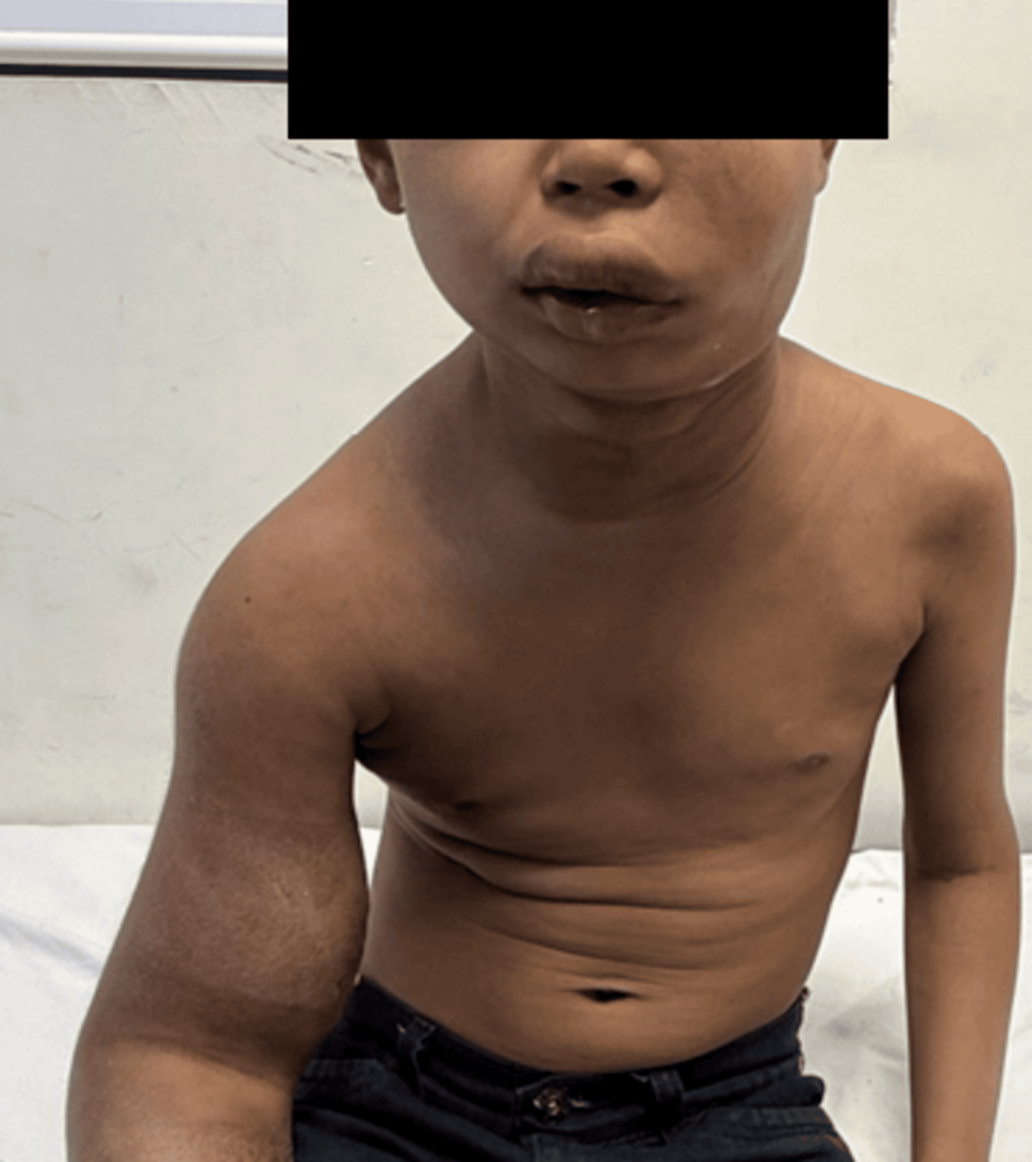
Diffuse asymmetrical swelling involving the right arm, forearm, right half of the anterior chest wall, neck, and left half of the face

**Figure 2:**
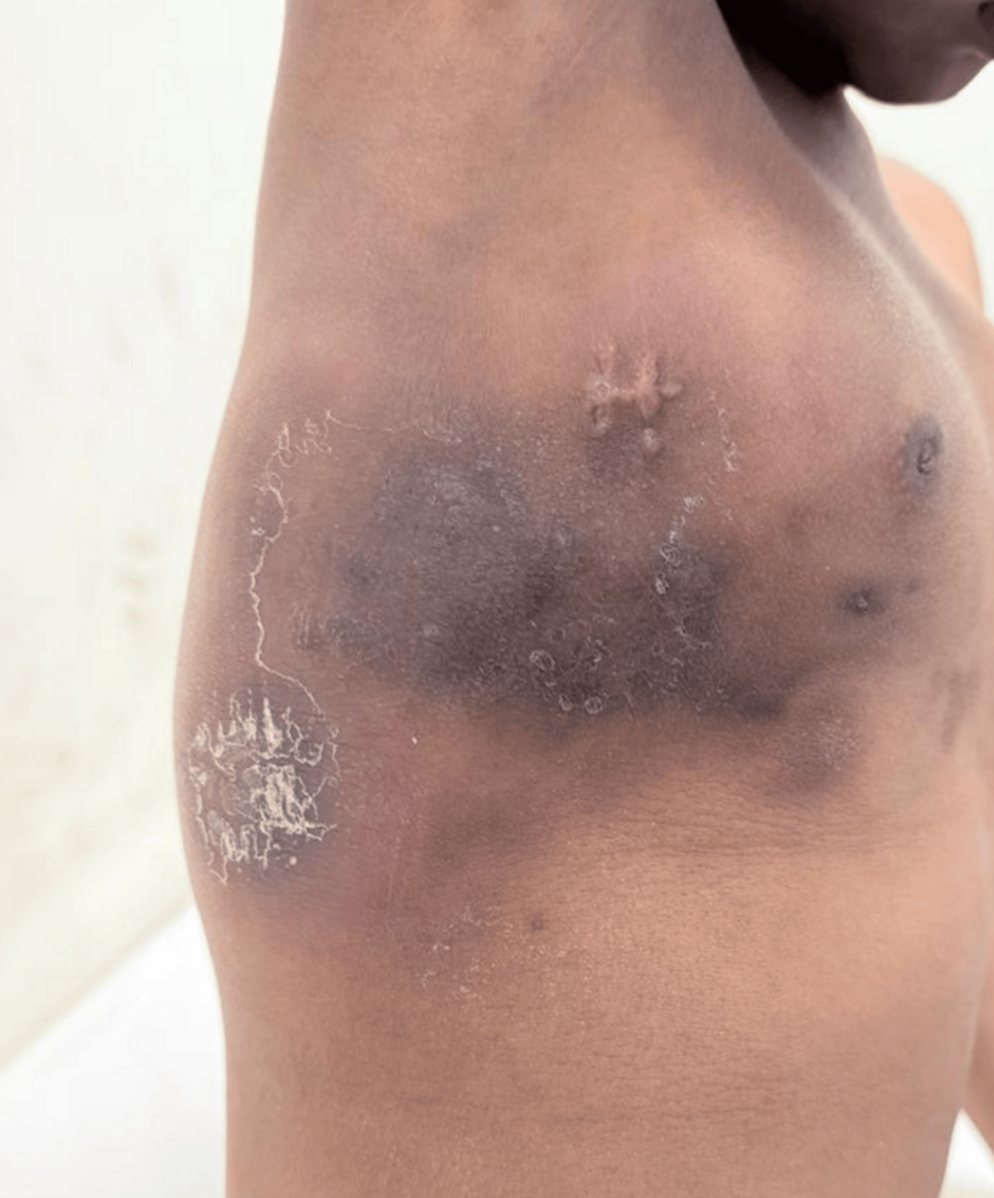
Diffuse skin thickening with erythematous plaques, induration, and excoriation along the right side of the chest wall

A nine-year-old boy from Telangana state, India, presented with a gradually progressive diffuse swelling for two months that was predominantly involving the right upper limb. The swelling was associated with multiple firm-to-hard, indurated subcutaneous plaques, some of which demonstrated mild erythema and hyperpigmentation. The lesions showed a contiguous distribution extending from the distal right forearm to the arm, shoulder, neck, anterior chest wall, and the right upper back. Induration and edema were appreciable over the right upper back (Figures 1, 2).

There was no associated pain, tenderness, fever, local rise in temperature, or bruit. Clinical examination demonstrated multiple palpable right axillary and cervical lymph nodes. There were no signs of neurovascular compromise.

**Figure 3:**
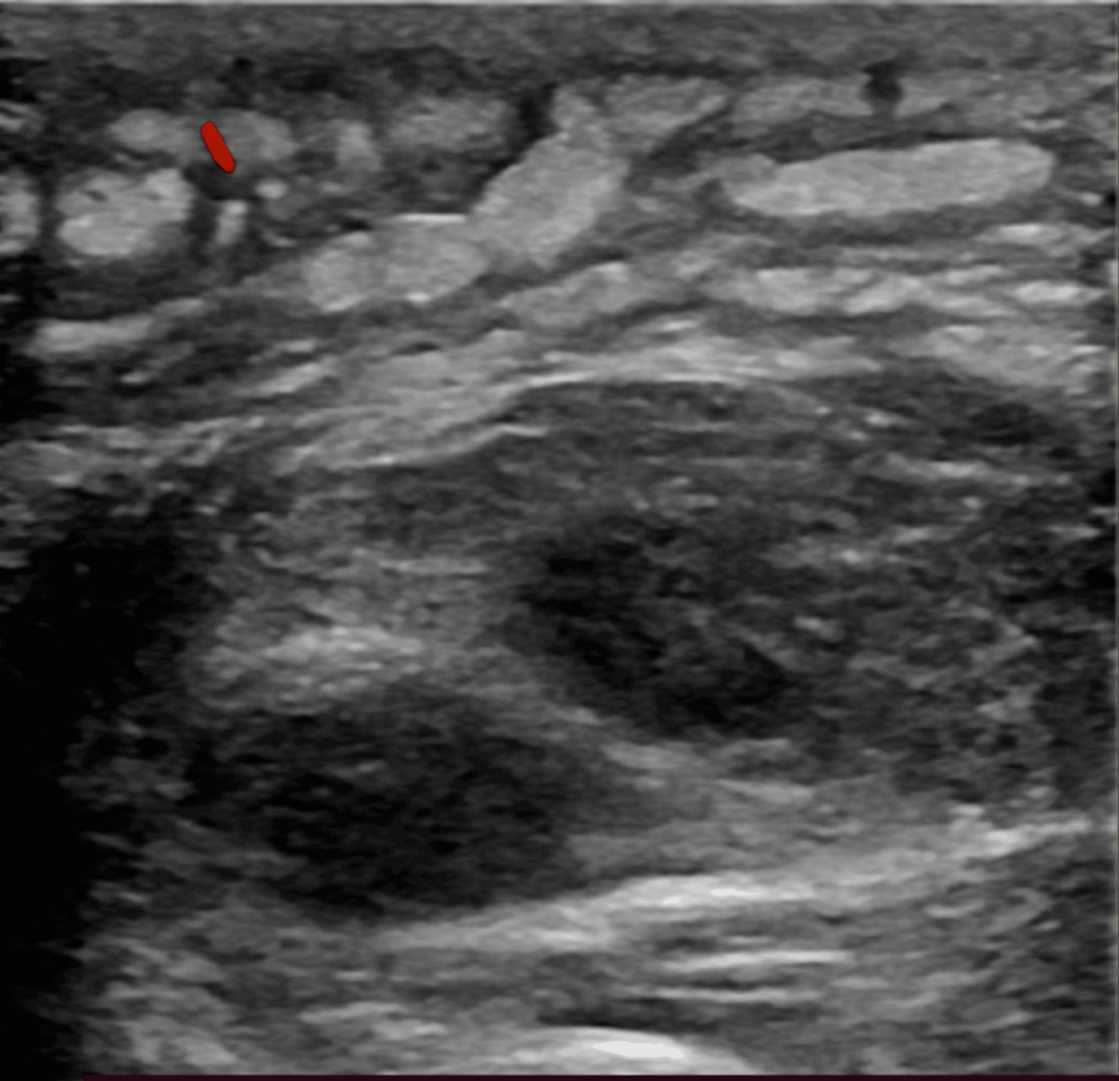
USG image at the level of the flexor aspect of the right arm, showing interstitial edema and thickening with a classical cobblestone appearance in the subcutaneous and intermuscular plane

**Figure 4:**
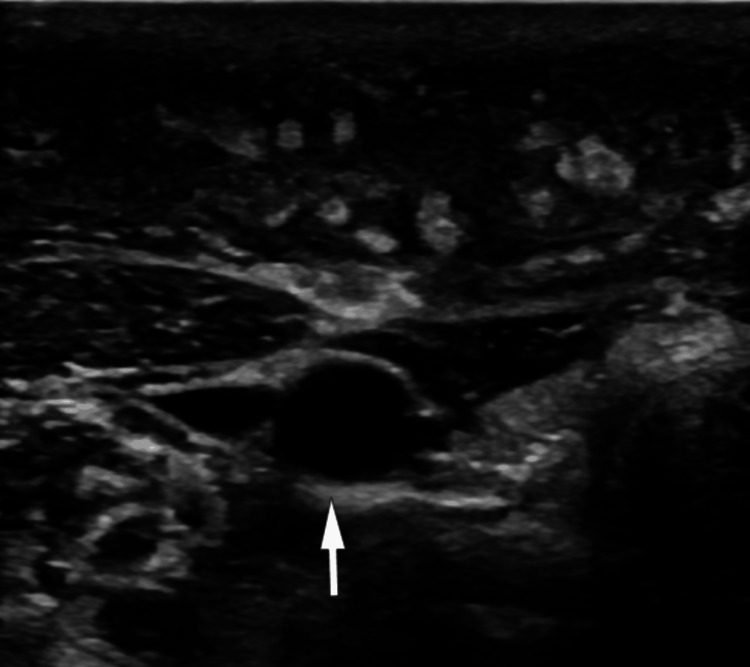
USG image shows normal vascular structures with fascial thickening and subcutaneous and intermuscular edema

Due to diffuse involvement in the subcutaneous plane, ultrasound was advised, which demonstrated diffuse subcutaneous soft-tissue thickening with linear edematous bands traversing through the subcutaneous fat and involving the right arm, right shoulder, right anterior chest wall, right posterior upper back, and right anterior aspect of the neck. Multiple ill-defined, nodular, granulomatous lesions were noted within the muscles, predominantly involving the biceps, deltoid, pectoralis, and brachialis muscles, associated with intramuscular edema (Figures 3, 4). No internal vascularity was demonstrated within these lesions on color Doppler ultrasound. There were no moving internal echoes, calcifications, or bony irregularities. A color Doppler study of the regional arteries and veins was within normal limits. Based on these findings, a chronic infective etiology with cellulitis and lymphoproliferative disorder was considered in the differential diagnosis. Contrast-enhanced magnetic resonance imaging (CE-MRI) was suggested to characterize the lesions further and to exclude a clinically suspected low-flow vascular or lymphatic malformation.

Laboratory investigations revealed anemia, thrombocytosis, and eosinophilia. These findings are summarized in Table 1.

**Table 1:** Hematological parameters at presentation

Parameter	Value	Normal Reference Range (as per age)
Hemoglobin	7.6 g/dL	11.5–15 g/dL
Total leukocyte count	23830 cells/µL	4500–13500 cells/µL
Platelet count	7.27 × 10⁵/µL	1.5–4.5 × 10⁵/µL
Absolute eosinophil count	0.68 × 10³/µL	0–0.5 × 10³/µL
Eosinophils	10.4%	1–6%

CE-MRI demonstrated multiple ill-defined areas of heterogeneous signal intensity involving the skin, subcutaneous tissues, muscles of the right upper limb, the right biceps, triceps, and deltoid muscles, the right upper chest wall, bilateral cervical regions, and the right latissimus dorsi muscle.

**Figure 5:**
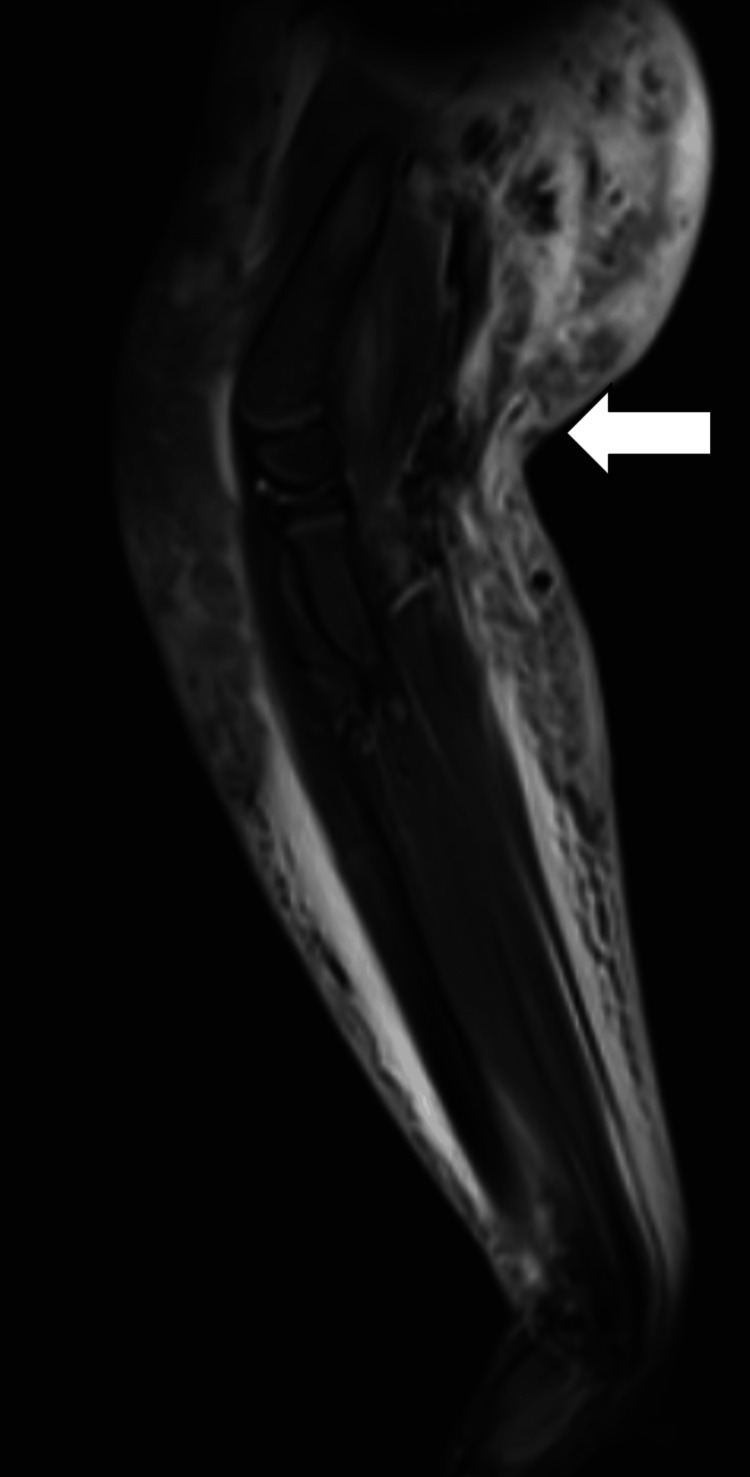
T2-weighted image of the right arm and forearm showing diffuse muscle thickening and subcutaneous interstitial edema (arrow) in all compartments of the right upper limb

**Figure 6:**
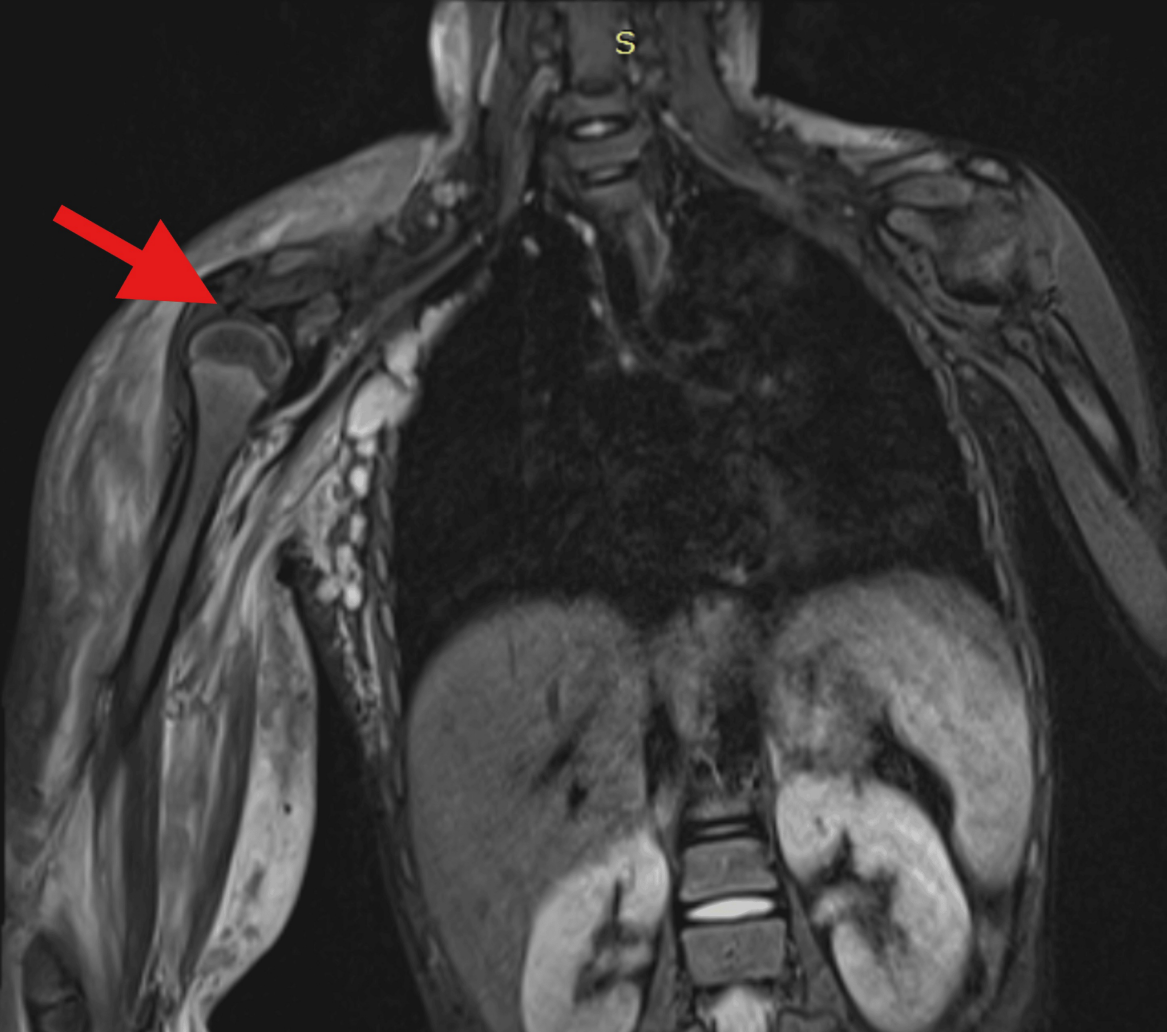
T2-STIR sequence revealing hyperintense edema in intermuscular and subcutaneous planes with intramuscular edema and enlarged axillary and pectoral lymph nodes T2-STIR: T2-weighted short-tau inversion recovery

**Figure 7:**
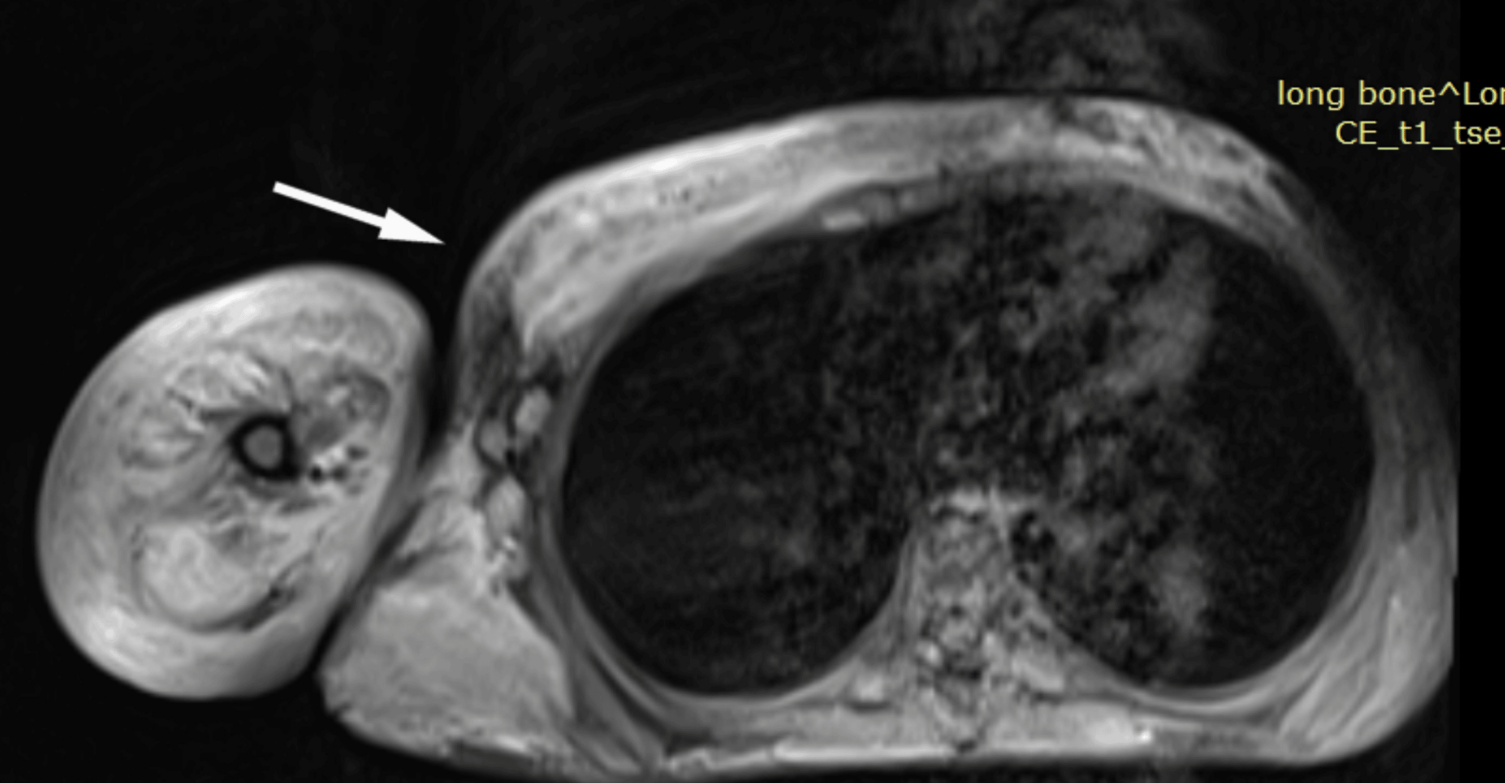
Post-gadolinium contrast T1 image showing mild enhancement in the involved region of the right arm and right chest wall

These areas were isointense on T1-weighted images and heterogeneously hyperintense on T2-weighted images with diffuse muscle thickening, interstitial edema, and heterogeneous post-contrast enhancement. No evidence of arteriovenous malformation or early arterial enhancement was identified on dynamic angiographic sequences in CE-MRI. Multiple enlarged bilateral axillary and cervical lymph nodes were noted without central necrosis (Figures 5-7). The imaging findings effectively excluded vascular malformations but remained nonspecific, perpetuating the diagnostic dilemma.

In view of a presumed case of chronic bacterial cellulitis, the patient was empirically started on oral amoxicillin-clavulanic acid and cotrimoxazole. Although there was an initial partial reduction in edema, the clinical course was marked by the appearance of new lesions over the right arm and chest within three months of initiating antibiotic therapy, along with progressive enlargement of the previously existing lesions. This lack of sustained response to antibiotics prompted further tissue diagnosis.

**Figure 8:**
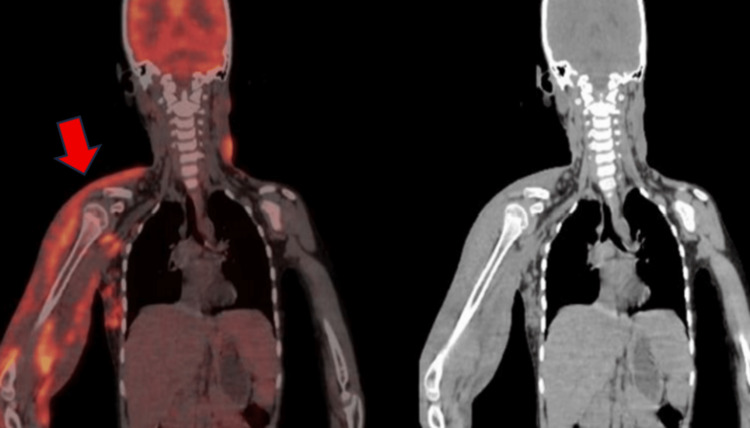
PET-CT demonstrated heterogeneously enhancing, metabolically active diffuse subcutaneous soft-tissue infiltration involving the right upper limb, thoracic wall, right cervical region, and left side of the face PET-CT: positron emission tomography-computed tomography

An ultrasound-guided targeted biopsy from the medial aspect of the right upper arm was performed, which revealed non-caseating granulomas with dense lymphocytic infiltrates and numerous eosinophils. Looking at the extensive nature of the disease, the possibility of subcutaneous lymphoproliferative malignancy needed to be ruled out; hence, a whole-body PET-CT was also performed. PET-CT demonstrated heterogeneously enhancing, metabolically active diffuse subcutaneous soft-tissue infiltration involving the right upper limb, thoracic wall, right cervical region, and left side of the face, with a maximum standardized uptake value (SUVmax) of 7.6 (Figure 8). These findings strongly suggested subcutaneous lymphomatous infiltration. Although multiple enlarged right axillary lymph nodes were noted, they did not demonstrate significant metabolic activity.

To further clarify the diagnosis, repeat targeted ultrasound-guided biopsies were performed on regions showing high metabolic activity on PET-CT, including the face and chest wall. Histopathological examination revealed dense lymphohistiocytic infiltrates with prominent eosinophils and plasma cells along with non-caseating granulomas. Multinucleated giant cells containing broad, focally septate fungal hyphae were identified. The fungal elements were highlighted on periodic acid-Schiff (PAS) and Gomori methenamine silver stains. No evidence of malignancy was detected. The histopathological findings were consistent with the diagnosis of subcutaneous zygomycosis.

**Figure 9:**
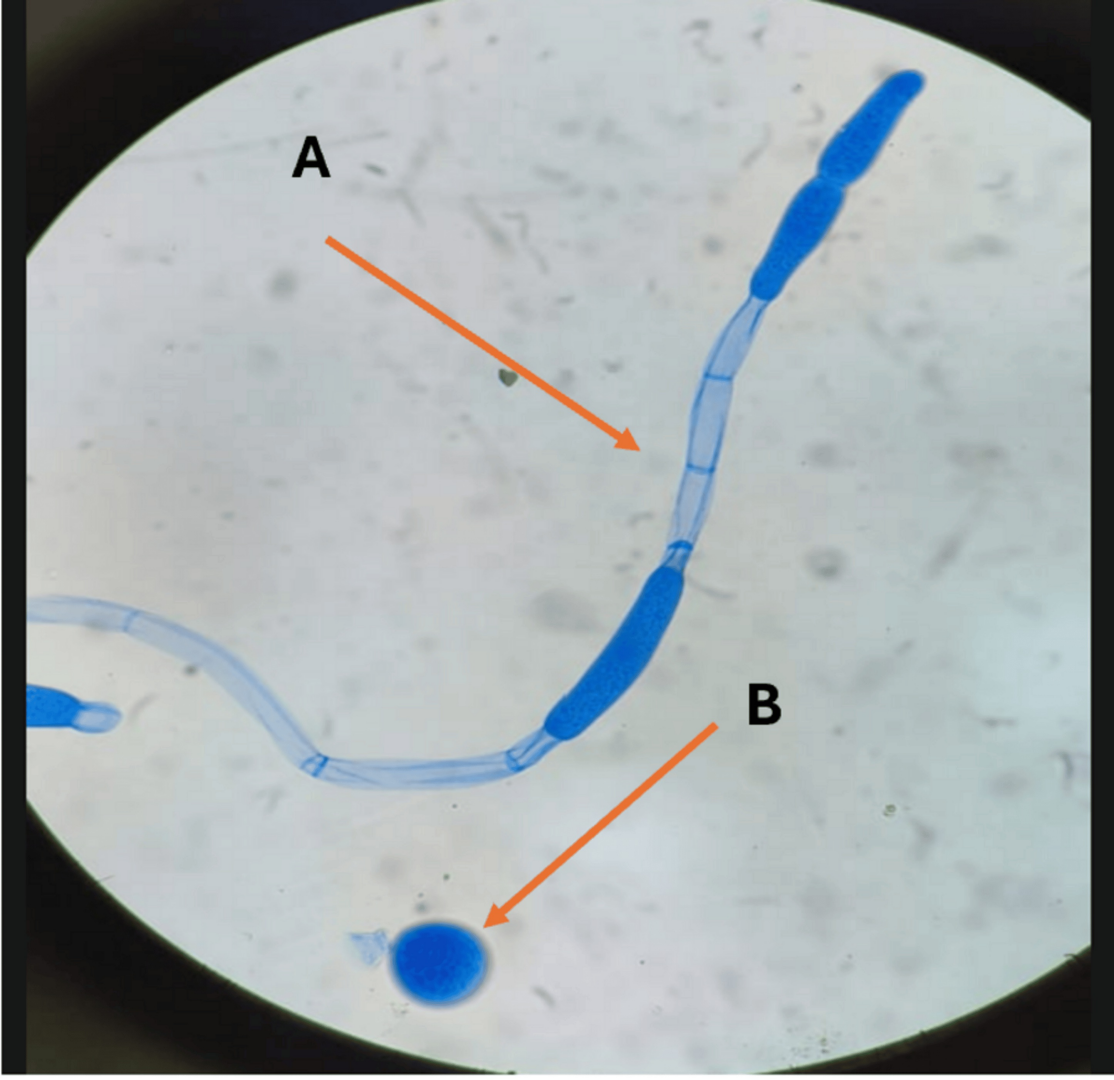
Lactophenol cotton blue mount showing broad ribbon-like septate hyphae (A) and developing gametangium or detached conidium (B)

**Figure 10:**
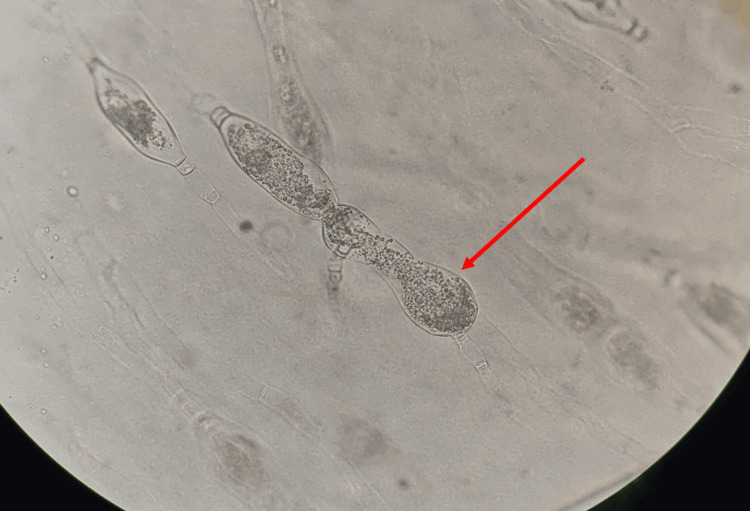
Slide culture revealed thick-walled zygospores with characteristic beak-like appendages

Subsequent microbiological evaluation of the biopsy specimen demonstrated growth of organisms belonging to the order Entomophthorales, confirming the diagnosis of basidiobolomycosis (Figures 9, 10).

**Figure 11:**
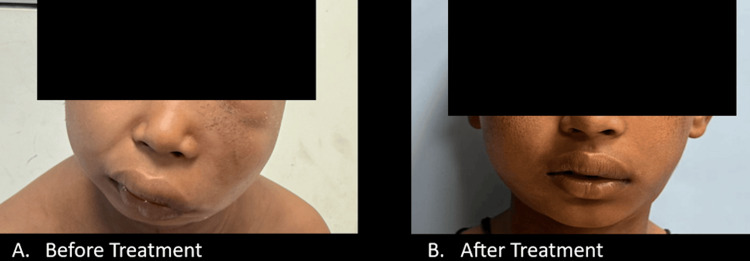
Evidence of a significant reduction in swelling and induration post-therapy with itraconazole

Following initiation of oral antifungal therapy with oral itraconazole at a dose of 130 mg once daily, adjusted to the child’s body weight of 22 kg, and follow-up at three months, significant clinical improvement was demonstrated. There was a marked reduction in the diffuse soft-tissue swelling with progressive resolution of the deformed face. The subcutaneous induration eventually softened, and the previously elicitable doughnut lifting sign was no longer present, indicating resolution of the subcutaneous infiltrative component and response to therapy (Figure 11).

## Discussion

Subcutaneous basidiobolomycosis is a well-documented diagnostic masquerader, frequently misinterpreted as inflammatory, neoplastic, or vascular pathology due to overlapping clinical and imaging features [5]. The present case exemplifies this dilemma, with sequential imaging raising suspicions of cellulitis, lymphoproliferative disease, and slow-flow vascular malformation before the definitive diagnosis was established.

Ultrasonography typically demonstrates diffuse subcutaneous thickening with edematous bands and hypoechoic granulomas, without abscess formation or internal vascularity. These findings are nonspecific but are significant when seen in conjunction with preserved normal Doppler vascular flow [2].

MRI further compounds the diagnostic challenge, as lesions appear ill-defined, T1 isointense, T2 hyperintense, and heterogeneously enhancing, closely mimicking soft-tissue infiltrative sarcoma, lymphoma, and inflammatory myositis [6]. Importantly, the absence of early arterial enhancement and lack of flow voids help exclude arteriovenous malformations and high-flow vascular lesions.

On PET-CT imaging, increased FDG uptake has been reported in inflammatory fungal lesions, often simulating malignant infiltration and leading to erroneous suspicion of lymphoma or sarcoma [6]. In the present case, elevated SUV values prompted repeat biopsies, underscoring the limitation of functional imaging in differentiating inflammatory infection from malignancy, especially in endemic regions.

A key differentiating clue repeatedly emphasized in the literature is the presence of peripheral eosinophilia, thrombocytosis, and chronic anemia in immunocompetent children with indolent, painless subcutaneous swelling [3]. Histopathology remains the diagnostic cornerstone, with identification of broad, sparsely septate fungal hyphae surrounded by eosinophilic material and non-caseating granuloma formation (Splendore-Hoeppli phenomenon), while fungal culture confirms the organism [7].

Radiologically, subcutaneous basidiobolomycosis must be differentiated from several mimics, including cellulitis, lymphoproliferative disorders, soft-tissue sarcomas, and low-flow vascular or lymphatic malformations. Unlike acute bacterial cellulitis, which typically presents with ill-defined subcutaneous edema with hyperemia and responds rapidly to antibiotic therapy, basidiobolomycosis shows chronic, progressive, infiltrative subcutaneous and intermuscular involvement without abscess formation or significant internal vascularity on Doppler imaging, often with a poor or absent response to antibiotics [2-5].

Soft-tissue sarcomas and rhabdomyosarcoma generally present as focal, mass-like lesions with infiltration of surrounding fat planes and frequent necrosis or hemorrhage, whereas basidiobolomycosis characteristically demonstrates diffuse plaque-like infiltration of the subcutaneous tissue, usually without destruction of underlying bone [6].

Lymphomatous involvement may show extensive soft-tissue infiltration with metabolically active lesions on PET-CT; however, basidiobolomycosis can demonstrate falsely elevated FDG uptake, thereby mimicking malignancy, with a lack of corresponding nodal metabolic activity serving as a potential clue toward an inflammatory etiology [6]. Low-flow vascular or lymphatic malformations are typically distinguished by the presence of serpiginous channels, phleboliths, fluid-fluid levels, or progressive contrast filling, features that are notably absent in basidiobolomycosis, which instead shows heterogeneous enhancement without early arterial filling or venous pooling on dynamic imaging [4]. Recognition of these imaging patterns, particularly when correlated with peripheral eosinophilia and a chronic indolent clinical course, is critical in guiding radiologists toward timely biopsy and accurate diagnosis. Ultrasonography-guided biopsy should be taken from evident granulomas or PET-CT avid lesions [1-4].

Effective treatment of basidiobolomycosis relies on early recognition of the disease, timely initiation of antifungal therapy, and management of any associated predisposing factors. Although many reported cases have required aggressive surgical debridement, the present case demonstrated a favorable clinical response with medical therapy alone. Treatment with itraconazole resulted in progressive and complete resolution of the disease, highlighting its efficacy as a conservative and safer therapeutic option and underscoring the potential to avoid extensive surgical intervention in appropriately diagnosed cases [7].

## Conclusions

This case highlights subcutaneous basidiobolomycosis as a rare but critical differential diagnosis for diffuse infiltrative soft-tissue lesions in children, particularly in endemic regions. From a radiological standpoint, recognition of nonspecific yet recurring imaging patterns across ultrasound, MRI, and PET-CT, when correlated with peripheral eosinophilia and lack of response to antibiotics, should prompt early tissue sampling. Awareness of this entity can prevent misdiagnosis, unnecessary oncologic workup, and delayed antifungal therapy, reinforcing the pivotal role of radiologists in resolving complex diagnostic dilemmas.
